# Editorial: Research integrity

**DOI:** 10.3389/frma.2023.1238504

**Published:** 2023-08-18

**Authors:** Teodora Konach, Rea Roje, Nicole Föger, Zoë H. Hammatt

**Affiliations:** ^1^Austrian Agency for Research Integrity, Vienna, Austria; ^2^University Hospital Split, Split, Croatia; ^3^World Conferences on Research Integrity Foundation, Amsterdam, Netherlands; ^4^Z Consulting LLC, Honolulu, HI, United States

**Keywords:** research integrity, research integrity education, early career researchers, PhD students, research integrity guidelines

PhD researchers and other early-career researchers (ECRs) play a vital role in research and contribute to the advancement of knowledge in all disciplines (Bernery et al., 2022; Pizzolato et al., 2023). Their experiences catalyze changes in the research system and reflect the current academic culture. PhD researchers provide new ideas and perspectives during the research process and they play an increasingly important role in the current production of scientific research—especially project-based research, which often requires a greater proportion of PhD researchers (Milojević et al., 2018; Boothby et al., 2022). In addition to the contributions they make, working on research projects also benefits junior researchers as well; such as the development of their research and interpersonal skills, as well as the advancement of their knowledge and expertise (Bernery et al., 2022; Pizzolato et al., 2023). Moreover, working on multidisciplinary and international collaborative research projects is a great opportunity for junior researchers to meet new colleagues, share experiences, and spread the collaborative network for further research endeavors.

As often analyzed and commented, researchers still in training frequently fulfill essential research tasks (Larivière, 2012; Bozeman and Youtie, 2017; Van Rooij et al., 2021). While this expanded role and responsibility of PhD researchers within the production of research knowledge may attract more talented emerging scientists and researchers to join the academic endeavor, despite the often short-term appointments, it is still unclear whether current recruitment strategies are aligned and reflects the long-term availability of tenured academic positions.

Little attention is also given to the cultural aspects of the rapid and novel developments within the research landscape, the costs of these transitions, and the fact that aligning individual values with the institutional environment is difficult to achieve in a very short period of time (Anonymous, 2016). The traditionally established transmission of practices, skills, and cultural behaviors in academia and research are significantly challenged by the new forms of collaboration, that very often offer (exclusively) project-based employment for the PhD students and other ECRs, that, in turn, only increases the turnover of the workforce, with a price of progressively reducing work stability. Moreover, a long-term, strategic vision of how academia and research careers are going to be portrayed in the years to come is not being sufficiently conceptualized yet. All these developments are generating enormous pressure on the youngest scientists and researchers in the system, but also on the more established researchers who may be without tenure or facing other challenges. Although progress and achievements related to research work may be gratifying, a sense of accomplishment does not come without stress and, quite often, situations where other areas of the life of PhD researchers are under strain (e.g., private, family, and socio-economic concerns (Powell, 2016; Wang et al., 2019; Woolston and O'Meara, 2019). In some situations, serious health issues, depression, and anxiety become a reality for junior researchers (Satinsky et al., 2021). The cause of stress is also the fact that this category of researchers is precariously positioned in research, fighting for a future career, permanent job position, and a confident place in academia (Cyranoski et al., 2011). However, their work and career advancement are not dependent only on themselves but rather influenced by many other, external, factors, such as the behavior, acts, support, and encouragement of their mentors and supervisors, but also the whole system of research which is today more than ever characterized by different pressures, such as the pressure to publish a lot of research or to publish in a high impact factor journal (Vatansever, 2020).

Further, the question is how the pandemic has impacted the career prospects and the wellbeing of PhD researchers and other ERCs. While some new trends of cooperation and doing research, such as remote work or work-from-home were reported as beneficial, younger researchers have experienced some significant limitations, like deficient channels to get more visibility for their research results, and obstacles to finding new collaborators and partners, due to mostly virtual or hybrid conferences, with fewer potential networking opportunities (Anonymous, 2020; Aydemir and Ulusu, 2020; Morens and Hammatt, 2021; Jamali et al., 2023). As quite often the PhD researchers are employed on fixed-term contracts, the time factor plays a crucial role in securing new co-operations, positions, and research projects that are mostly being generated through networking, establishing personal contacts, and interacting with established members of academic or research settings.

Another question is how much PhD researchers know about research integrity and whether they received any training in this area, and further, whether the training they received enabled them to acquire the knowledge and skills to conduct research responsibly and also to spot and respond to research misconduct and other breaches of research integrity (Haven et al., 2022). To make it easier for PhD researchers, several adjustments in the current academic environment should be made, with an emphasis on research integrity. First, there should be some kind of incentive, for example by major funders and research organizations, to create more permanent positions for junior researchers to be able to continue their careers. Second, stakeholders in the system of science (research performing organizations, funding organizations, and professional societies) should invest more efforts in reducing the competition pressure and changing the evaluation requirements in which the focus from the number of publications would be switched to the quality of publications and other criteria not related to the quantity of research work. Moreover, there should be more initiatives related to open science, and translating open science practices into responsible research should be more valued.

In this Research Topic on research integrity, we address various issues and diverse perspectives ranging from educating PhD researchers on research integrity, promoting research integrity among the academic community, establishing research integrity policies within research organizations, to implementing open science practices and investigations of research misconduct conducted by researchers of various research ranks.

Martinez-Campos presents tips for teaching research integrity successfully to PhD researchers. The author provides 10 tips based on 20 years of experience in teaching research integrity to PhD researchers. It is essential that mentors and supervisors ensure adequate and proactive training and education on research integrity for junior researchers. Besides suggestions for those who educate PhD researchers, the responsibilities of research organizations are also emphasized across the scientific community. Cao et al. point to the suboptimal research integrity policies provided by research organizations. This makes it even harder for PhD researchers to know how to apply responsible research practices in their everyday research and avoid and report research misconduct or detrimental research practices. Policies and guidance for research integrity should be updated regularly and tailored to the researchers' needs. Stavale et al. discuss the need for clarity when it comes to research integrity policies and investigations of research misconduct which are both important for ensuring and fostering high-quality and trustworthy research. Further, Lindermann and Haberlein provide their vision on how to adopt research integrity perspectives to support researchers and other stakeholders in operationalizing open science principles. This article outlines how research integrity may help researchers, including PhD researchers, understand the importance of open science and how to apply it in practice. Zhang, relating to a sense of being proactive and transparent in promoting research integrity, offers several suggestions for better implementation of research integrity. These include team building, capacity building, and researcher empowerment which is very important for PhD researchers just stepping into the academic world.

The academic community should ensure that the voice of PhD researchers and other ERCs is heard. Even if they do not have a lot of experience, PhD researchers bring new knowledge and approaches, fresh and diverse perspectives, talent, and enthusiasm to research.

As such, listening carefully and responding to their concerns and needs can help ensure that responsible research practices are implemented from the very start of their careers, thereby helping to enhance the entire research culture in which they work. Although the number of PhD researchers is on the rise, influenced by the greater number of funded research projects and opportunities, a closer look at this shows that only half of these researchers pursue more permanent positions related closely to their PhD and that the number of opened positions for further career is way smaller than the number of PhD students (National Academies, of Sciences, Engineering, and Medicine, 2017). This leaves the question of whether PhD researchers and their enthusiasm are used only as the working force on the projects with only a few of them having the opportunity for a prospective career in academia. At the same time, voices are raised repeatedly over major concerns that the most talented scientist and researchers will not stay in academia (Powell, 2016). Finally, taking all these developments into consideration, it might be worth asking if the PhD researchers and other ECRs are truly “the future” of academia? As also discussed by Christian et al. (2021), only 51.0% of their survey respondents indicated satisfaction with their workplace, a study that is echoing previous research done (Johnsrud and Rosser, 2002; Smith, 2020).

In this editorial, we seek to give a voice to PhD researchers and other ERCs in different parts of the world. They are asking and attempting to answer questions related to their place, role, and wellbeing within the current research system. A more holistic discussion is sorely needed. And PhD researchers need to be heard. Through their insights, we seek to expand the discussion on career prospects, research environment, and culture, mentorship and supervision practices, training in research integrity, and the acquisition of skills and knowledge in good research practice. In this way, we envisage a more balanced conversation. We believe this can help pave the way for early career researchers as they participate in efforts to reimagine and reshape their future along with the future of the global research endeavor.

## Author contributions

TK and RR developed the idea for the manuscript and contributed to the conceptualization, design, writing, and editing the manuscript equally. NF and ZH contributed to the design, writing, and editing the manuscript. All authors contributed to the article and approved the submitted version.
